# Predicting Chemotherapeutic Response for Far-advanced Gastric Cancer by Radiomics with Deep Learning Semi-automatic Segmentation

**DOI:** 10.7150/jca.46704

**Published:** 2020-10-18

**Authors:** Jing-wen Tan, Lan Wang, Yong Chen, WenQi Xi, Jun Ji, Lingyun Wang, Xin Xu, Long-kuan Zou, Jian-xing Feng, Jun Zhang, Huan Zhang

**Affiliations:** 1 Department of Radiology, Ruijin Hospital, Shanghai Jiao Tong University School of Medicine, Shanghai, China; 2 Department of Oncology, Ruijin Hospital, Shanghai Jiao Tong University School of Medicine, Shanghai, China; 3 Haohua Technology Co., Ltd, Weihai International Group Building, No. 511 Weihai Road, Shanghai, China

**Keywords:** Delta radiomics, far-advanced gastric cancer, deep learning, semi-automatic segmentation, dual-energy computed tomography

## Abstract

**Purpose:** To build a dual-energy computed tomography (DECT) delta radiomics model to predict chemotherapeutic response for far-advanced gastric cancer (GC) patients. A semi-automatic segmentation method based on deep learning was designed, and its performance was compared with that of manual segmentation.

**Methods:** This retrospective study included 86 patients with far-advanced GC treated with chemotherapy from September 2016 to December 2017 (66 and 20 in the training and testing cohorts, respectively). Delta radiomics features between the baseline and first follow-up DECT were modeled by random forest to predict the chemotherapeutic response evaluated by the second follow-up DECT. Nine feature subsets from confounding factors and delta radiomics features were used to choose the best model with 10-fold cross-validation in the training cohort. A semi-automatic segmentation method based on deep learning was developed to predict the chemotherapeutic response and compared with manual segmentation in the testing cohort, which was further validated in an independent validation cohort of 30 patients.

**Results:** The best model, constructed by confounding factors and texture features, reached an average AUC of 0.752 in the training cohort. Our proposed semi-automatic segmentation method was more time-effective than manual segmentation, with average saving-time of 11.2333 ± 6.3989 minutes and 9.9889 ±5.5086 minutes in the testing cohort and the independent validation cohort, respectively (both p < 0.05). The predictive ability of the semi-automatic segmentation was also better than that of the manual segmentation both in the testing cohort and the independent validation cohort (AUC: 0.728 vs. 0.687 and 0.828 vs. 0.749, respectively).

**Conclusion:** DECT delta radiomics serves as a promising biomarker for predicting chemotherapeutic response for far-advanced GC. Semi-automatic segmentation based on deep learning shows the potential for clinical use with increased reproducibility and decreased labor costs compared to the manual version.

## Introduction

Gastric cancer (GC) is a considerable health burden worldwide, accounting for more than one million newly diagnosed individuals and appropriately 783,000 deaths in 2018 1. Despite radical resection being considered the only curable treatment for GC, nearly two-thirds of patients present with unresectable, locally advanced or metastatic disease with poor outcomes when initially diagnosed 2. For those patients, systemic chemotherapy is recommended to prolong overall survival from 4 months to 12 months compared with the best supportive care 3. Despite improved survival, the standard regimen of systemic chemotherapy is still controversial, and the reported rate for tumor response is less than 40% 3,4. With the coexistence of intratumor heterogeneity 5 as well as underlying mechanisms of resistance to chemotherapy in GC 6, it seems that evaluating the chemotherapeutic response at timepoints after a defined follow-up time may be too late, especially for long cycles of chemotherapy regimens. Patients not converting to appropriate treatment strategies may endure unnecessary chemotherapeutic toxicity. Therefore, it would be more beneficial to predict treatment response at early stages of chemotherapy courses before certain chemotherapy resistance occurs.

Response Evaluation Criteria in Solid Tumors (RECIST) version 1.1 is accepted as the set of criteria for evaluating the therapeutic response for solid tumors. However, the RECIST criteria are applied to evaluating the treatment response based on the current therapeutic timepoint instead of predicting the response in advance, which may delay the time for intervention. In addition, due to the movement of the stomach and the irregular and nonspherical shape of lesions in the stomach wall, measuring the longest tumor diameter as the only criterion is difficult to reproduce for the purposes of making predictions 7. Given the current drawbacks, it is crucial to improve the efficacy in evaluating the chemotherapeutic response.

Radiomics is a promising field for providing insight into tumor heterogeneity by noninvasively extracting and analyzing massive numbers of quantitative features beyond what can be observed by the naked eye 8. Compared with biopsy specimens, radiomics can noninvasively and repeatedly sample the heterogeneity of and screen the entire tumor in succession during chemotherapy courses, which may provide interferences in advance of any ineffectiveness or deterioration. Previous publications have reported the predicting value of radiomics on the prognosis for locally advanced GC 9-12. However, those studies focused on using radiomics to evaluate treatment response or predict survival for local advanced GC treated with neoadjuvant chemotherapy or postsurgical chemotherapy based on images pre-treatment. As mentioned above, the resistance to chemotherapy drugs may result from individual differences, making radiomics analysis based only on pretreatment or posttreatment less convincing. Delta radiomics, defined as the changes in features according to the dynamic process that occur during therapeutic courses makes possible personalized predictions for chemotherapeutic benefits 13. Besides, dual-energy computed tomography (DECT) is a promising modality for stomach imaging due to its potential to provide a large amount of pathophysiological information 14 and no prior studies have investigated the role of DECT in GC using radiomics, except for one that applied DECT radiomics to predict lymph node metastasis 15.

Volumes of interest (VOIs) are recommended in radiomics research 16. Given intratumor heterogeneity, delineation of only one slice may not provide as much information as delineation of the entire tumor. In addition, compared with one-dimensional measurements, the tumor volume could more accurately depict the dynamic changes in tumor burden and morphology during treatment 17. The combination of tumor morphology and delta radiomics, showing tumor burden and heterogeneity, respectively, has the potential to provide a large amount of information for predicting the chemotherapeutic response. Nevertheless, manually delineating the entire tumor slice by slice, especially for thin-section CT images, is observer inconsistent and time consuming 7. For delta radiomics, which requires repeated delineation at different timepoints, highly efficient segmentation is urgently needed. Deep learning, a multilayer stack of neural networks, discovers intricate structures from big data and has been widely used for the segmentation of biological images for its accuracy and efficiency 18. In the field of GC, studies have mainly concentrated on modalities using endoscopy images and pathological sections 19-21. Due to the movement and irregular shape of GCs and background noise and the complexity of the abdomen, to our knowledge, no studies have delineated GC using deep learning on CT to date.

In this study, we aimed to build a DECT delta radiomics model to predict the therapeutic response of far-advanced GC patients during continuous intermittent chemotherapy, which may have potential benefits for timely intervention and adjustment of treatment strategies in advance. In addition, we aimed to develop and validate a semi-automatic segmentation method using a modified V-net deep learning algorithm for far-advanced GC and then compare the performance of the predictive value for the chemotherapeutic response with that of manual segmentation.

## Materials and Methods

### Patients

This retrospective study was approved by our local ethics committee, and informed consent was waived. Data from patients with far-advanced GC during the period from September 2016 to December 2017 were collected. Far-advanced GC was defined as recurrent GC or GC with peritoneal or distant metastasis or tumors surrounding major vessels on CT examination (cT_4a~b_N_x_M_0~1_). The inclusion criteria were as follows: 1) histologically confirmed primary stomach adenocarcinoma; 2) no prior history of chemotherapy or radiotherapy; 3) administration of at least 6 cycles of chemotherapy; and 4) availability of at least three DECT examinations obtained before chemotherapy within one week and the first and second follow-up DECT images.

**Figure 1:**
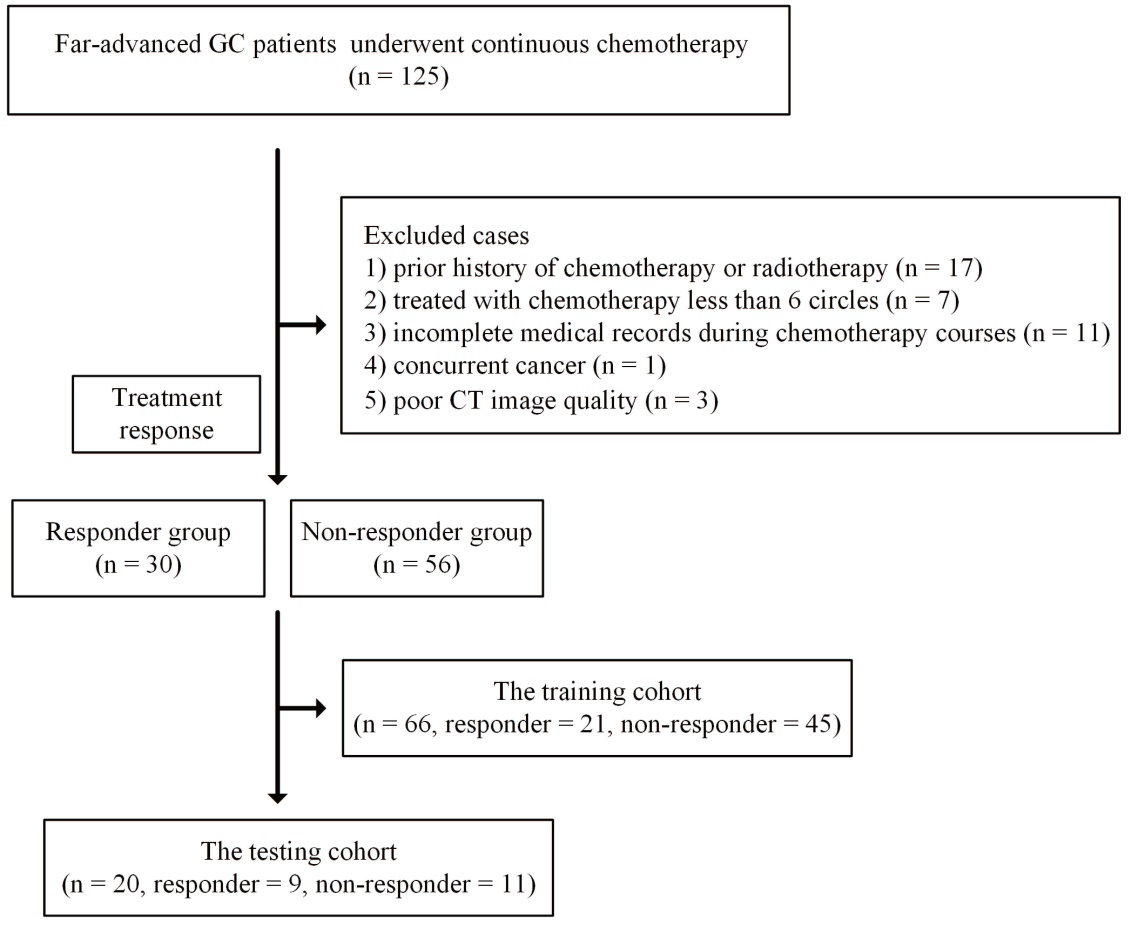
Flowchart for included patients in the training and testing cohorts. Abbreviation: CT: computed tomography; GC: gastric cancer.

The exclusion criteria were as follows: 1) incomplete medical records during chemotherapy courses or poor CT image quality due to artifacts; 2) concurrent cancer; and 3) intolerance to chemotherapy due to poor performance status. Detailed information is presented in Figure 1.

Treatment response was evaluated after the second follow-up CT examination based on RECIST 1.1 22. Patients with complete response and partial response were classified as the responder group, while patients with stable disease and progressive disease were classified as the nonresponder group 23. Follow-up CT was performed every 2~3 months after chemotherapy. The imaging protocol was described in our previous study 24 and is presented at Doc S1.

Finally, a total of 86 patients who underwent at least three CT scans were enrolled in this study. These 86 patients were further randomly split into training (66 patients, 21 responders and 45 nonresponders) and testing (20 patients, 9 responders and 11 nonresponders) cohorts.

### Chemotherapy regimen

Platinum or taxane-based combination chemotherapy was the main regimen applied in this study, including S-1 plus oxaliplatin (SOX regimen) and S-1 plus docetaxel (PS regimen), accounting for a total of 53 patients among the 86 in the study. For the training cohort, 14 and 28 patients adopted the SOX and PS regimens, respectively. For the testing cohort, the numbers of patients who underwent SOX and PS were seven and four, respectively.

For the SOX regimen, patients received S-1 at a dose of 60 mg/m^2^ orally twice daily from days 1 to 14. Oxaliplatin was administered intravenously for 2 hours at 130 mg/m^2^ on day 1. Cycles were repeated every 21 days.

For PS regimen, patients received S-1 at a dose of 60 mg/m^2^ orally twice daily from days 1 to 14. Docetaxel was administered intravenously for 1 hour at 40 mg/m^2^ on day 1. Cycles were also repeated every 21 days.

Chemotherapy toxicity was evaluated after every cycle, and treatment was given for at least six cycles unless disease progression, intolerable toxicity or death occurred.

### Performance of the CT delta radiomics model by manual segmentation

#### Manual segmentation

Manual segmentation was considered as the reference and was performed on the delayed phase images. Two radiologists (L.W. and J.T., with five and six years of experience in abdominal imaging, respectively), blinded to the pathological and clinical outcomes, delineated the 3D region of interest (ROI) with ITK-snap (version 3.6.0) along with the edge of the tumor slice by slice on axial images. Partial volume effects were avoided by omitting the ROIs from the initial and final slices.

#### Radiomics features

Open source pyradiomics packages implemented in Python were used to extract radiomics features from the CT images, which complies with the Image Biomarker Standardization Initiative 25,26. The extracted features included shape-based features (n=18), first-order histogram features (n=14), and second-order histogram features (texture features, n=75). The texture features consisted of Gray Level Co-occurrence Matrix (GLCM), Gray Level Run Length Matrix (GLRLM), Gray Level Size Zone Matrix (GLSZM), Gray Level Dependence Matrix (GLDM), and Neighboring Gray Tone Difference Matrix (NGTDM). For each patient, a total of 107 features were extracted from the segmented region of interest on each CT image. Feature information is presented in Table S1. Delta radiomics features were calculated by related difference from ROIs between baseline and the first follow-up CT examinations.

#### Clinical features for confounding factors

In addition to the delta radiomics features at baseline and the first follow-up DECT, we also considered four clinical features that could be potential confounding factors. These four features were the max length of the ROIs (mm) from the baseline DECT images, the max length of the ROIs (mm) from the first follow-up CT images, the time interval (days) between the baseline CT examination and the first follow-up CT examination, and the time interval (days) between the baseline CT examination and the second follow-up CT examination. The four confounding factors were then analyzed independently and combined with the radiomics features.

#### Optimal feature subset and modeling by manual segmentation

The random forest (RF) method was used to predict the therapeutic response for its simplicity and effectiveness 27. To select a suitable subset of features for classification, we performed the analysis in two steps: 1) evaluating the performance of the four confounding factors, all 107 delta radiomics features and the combination of both subsets; 2) evaluating subgroups of the delta radiomics features grouped into three categories and the combination of the confounding factors and the three new subgroups of delta radiomics features.

Therefore, a total of nine feature subsets were constructed, including five single and four combination feature subsets. They consisted of the subset of confounding factors, all delta radiomics features, a combination of confounding factors and all delta radiomics features, first-order features, a combination of confounding factors and first-order features, shape features, a combination of confounding factors and shape features, texture features and a combination of confounding factors and texture features. For simplification, we renamed each feature subset as Subset 1 to Subset 9 according to the above order (Table S2).

To explore which feature subset is more effective in classification, we perform 10-fold cross-validation on the training cohort. Therefore, the training set was further divided into ten folds, of which eight were used for training in turn, and the other two folds were used for validation and testing separately. We repeated the 10-fold cross-validation ten times, and we recorded the average area under the curve (AUC) and accuracy (ACC) and the corresponding standard deviation at each time point. Finally, the best CT delta radiomics model was chosen for comparison with the model developed by semi-automatic segmentation.

#### Sensitivity analysis for different chemotherapy regimens

Given the different chemotherapy regimens used in this study, the therapeutic effect may have nonnegligible variation among the different chemotherapy drugs. To verify this influence, we also conducted a sensitivity analysis between chemotherapy regimens and radiomics features (the nine feature subsets described above) in the training cohort. The names of feature subsets were appended with an “S” or a “P” for the SOX and PS regimens, respectively. For example, Subset 1 for all patients was named SSubset 1 for patients treated with the SOX regimen (Table S2). However, due to the limited size, the analysis for the testing cohort was not performed. We selected the two most commonly used chemotherapeutic regimens (SOX and PS regimens) in our study. There were 14 and 28 patients who received the SOX and PS regimens, respectively, accounting for nearly 2/3 of the patients (42/66). The performance of the delta radiomics model in each chemotherapy regimen was evaluated with the classification metrics applied above.

### Semi-automatic segmentation using deep learning

Manual segmentation is time-consuming; therefore, we developed a deep learning model for semi-automatic segmentation of the GC region. The model was trained on the training cohort and evaluated on the testing cohort.

To further validate the performance of the semi-automatic segmentation and compare with manual segmentation, we also recruited a group of patients as an independent validation cohort from January 2018 to July 2018. Inclusion and exclusion criteria were consistent with the Patients section. Finally, 30 patients were enrolled (11 responders and 19 nonresponders).

#### V-Net segmentation model

A volumetric, fully convolutional neural network referred to as V-Net 28 was adopted to perform segmentation on the 3D image. The V-Net architecture is the 3D version of U-Net 29; specifically, it introduces 3D convolutional and residual models (Figure S1) in the architecture. The entire network consists of a compression path that gradually reduces a 256×256×32 input image to an 8×8×4 representation and a decompression path, then gradually upsampled the representation to a 256×256×32 output while increasing the number of channels from 1 to 256 and then decreasing the number to 32.

The different stages of the network were operated at different resolutions. Each stage comprised one to three convolutional layers, and each convolution used 5×5×5 volumetric kernels applied with a stride of 1. Along the compression path, convolution with 2×2×2 voxel kernels applied with a stride of 2 was used at the end of each stage to reduce the resolution. Conversely, along the decompression path, 2×2×2 deconvolution with a stride of 2 was applied to decompress the data to a larger size. The features extracted were forwarded from the early stages of the left part of the network to the right part to improve the quality of the final contour prediction. At the end of the network, 1×1×1 convolution, which produces an output of the same size as the input, was applied to compute the two feature maps, and softmax was applied to assign probabilistic segmentations of the foreground and background regions. In our study, we applied ReLU instead of the original PReLu throughout the network.

#### Training details for semi-automatic segmentation

The V-Net deep learning network was trained with the training cohort and validated with the test cohort. Deep learning algorithms usually require a large amount of training data. Because the ground truth annotations of medical images need to be generated manually by experts, annotated medical images are not easily obtained. Therefore, to obtain robustness and an increased precision for the test dataset, we applied image rotation and warping to augment the original training dataset.

In our study, for each 3D CT image in the training data, the rotation angle in the x or y direction was randomly selected from {-1, 0, 1} radians, and the rotation angle in the z direction was randomly selected from [-18, 18]. After augmentation, there were a total of 3309 3D CT images in the training data, and we further split these training images into a training cohort (95%) and a validation cohort (5%).

Prior to training our model, we applied scipy.ndimage.zoom to scale the 512×512×z CT images (the whole dataset, including training, validation and testing images) to 256×256×z. Voxel values out of the range of [-200, 200] were clipped, and the remaining values were normalized to [0, 1]. For each 3D CT image, a randomly selected, continuous 32 layers of images were chosen as a training instance for the neural network input.

### Evaluation of the semi-automatic segmentation model and the radiomics model on the testing cohort and the independent validation cohort

In the testing cohort, the semi-automatic segmentation results were compared with the manual segmentations, and the performance was evaluated with the Dice similarity coefficient. The semi-automatic segmentation results were further revised manually to form the semi-automatic segmentation results. The time consumption for annotating the segmentation from scratch based on the semi-automatic segmentation results was recorded and compared. The indices were further validated in the independent validation cohort.

**Figure 2:**
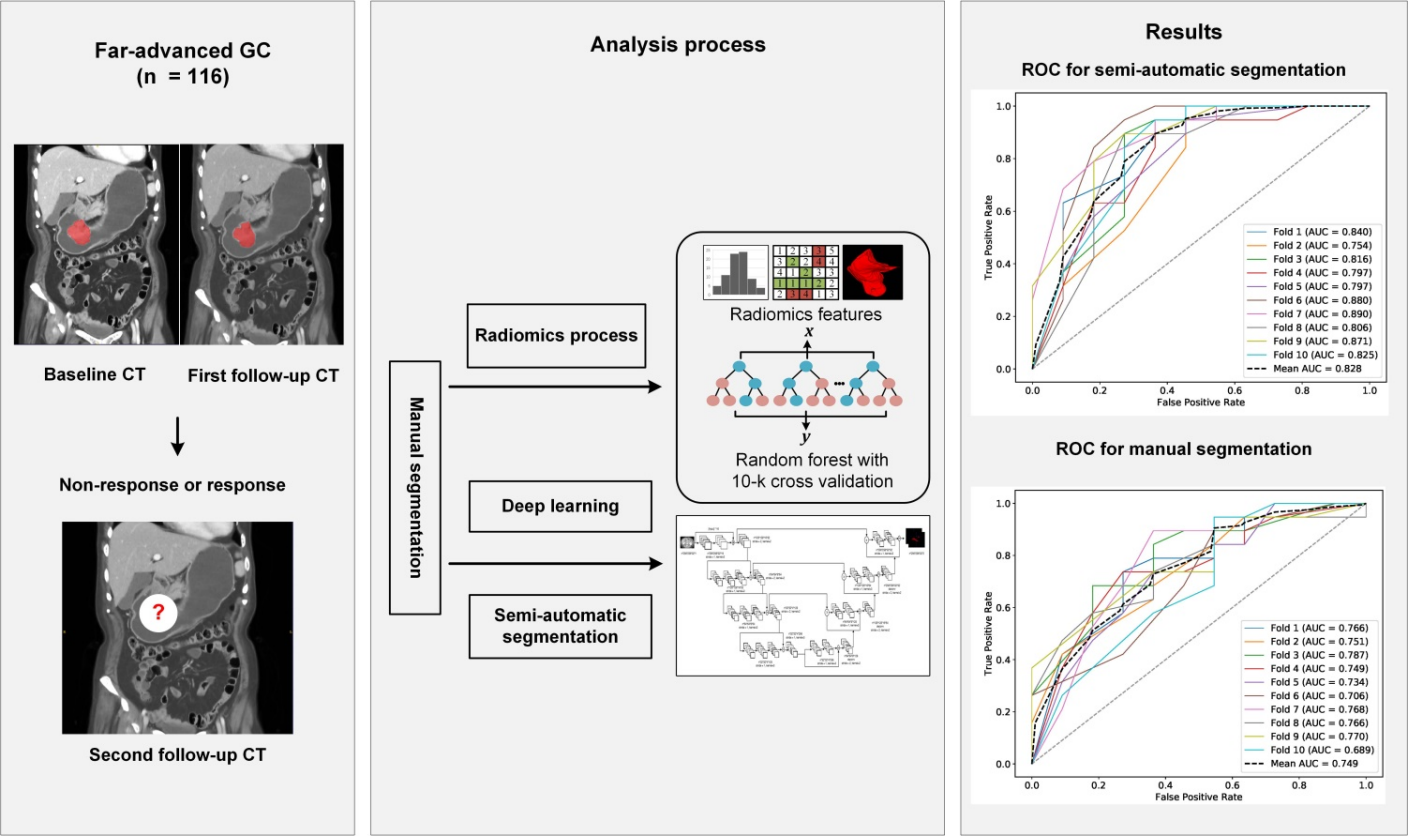
The workflow of this study. Abbreviation: CT: computed tomography; GC: gastric cancer; ROC, receiver operating characteristic curve.

RF radiomics models for predicting the chemotherapeutic response for far-advanced GC were built based on all data from the training cohort. Features were extracted from the manual segmentations and semi-automatic segmentations. The performance of the radiomics models was compared between the two segmentation methods on both of the testing cohort and the independent validation cohort. Due to the randomness property of the random forest model, we repeated the whole process 10 times to obtain a more solid result. Flowchart of this study is shown in Figure 2.

### Statistics

Statistical analysis was conducted using R (Version 3.4.1). Clinical characteristics were analyzed according to the distribution of the variable; that is, continuous variables are shown as the means or medians and compared using independent t tests or Wilcoxon rank-sum tests based on their distributions. Categorical variables were measured as proportions and were compared using chi-squared tests or Fisher's exact test. Significant differences were considered at p < 0.05.

## Results

### Clinical characteristics

The clinical characteristics of the enrolled patients are presented in Table 1. Based on the first follow-up CT examination, 61 and 25 patients' demonstrated nonresponse and response, respectively, compared with the baseline status. Based on the second follow-up CT examination, 56 patients showed nonresponse, while 30 patients showed response when compared with the first follow-up CT. No bias was found between these two cohorts in terms of treatment response (*x*^2^ = 1.174, p = 0.279). Of all medical records, only gender was at the borderline level of statistical significance in the training cohort (*x*^2^ = 4.002, p = 0.045). No clinical feature was found to be significantly different in the testing cohort. In the independent validation cohort (male 20; female 10), mean age was 56.3 ± 13.4 years. No difference was found in the independent validation cohort for gender and age (p = 0.893 and 0.715, respectively).

### Optimal feature subset and modeling by manual segmentation

The performance of the confounding factors alone (Subset 1) in predicting the chemotherapeutic response had a mean ACC and AUC of 57.4% and 0.478, respectively. In contrast, the independent delta radiomics features (Subset 2) showed better predictive value using random forest, with mean ACC and AUC values of 72.1% and 0.736, respectively. When confounding factors with delta radiomics features were incorporated, the performance of Subset 3 improved (mean ACC and AUC of 73.0% and 0.749, respectively). When subgroup analysis was performed, the best radiomics subset was the combination of confounding factors and texture features (Subset 9), which was slightly improved compared with Subset 3 (mean ACC of 73.8% and mean AUC of 0.752). Detailed information is shown in Table S3.

**Figure 3:**
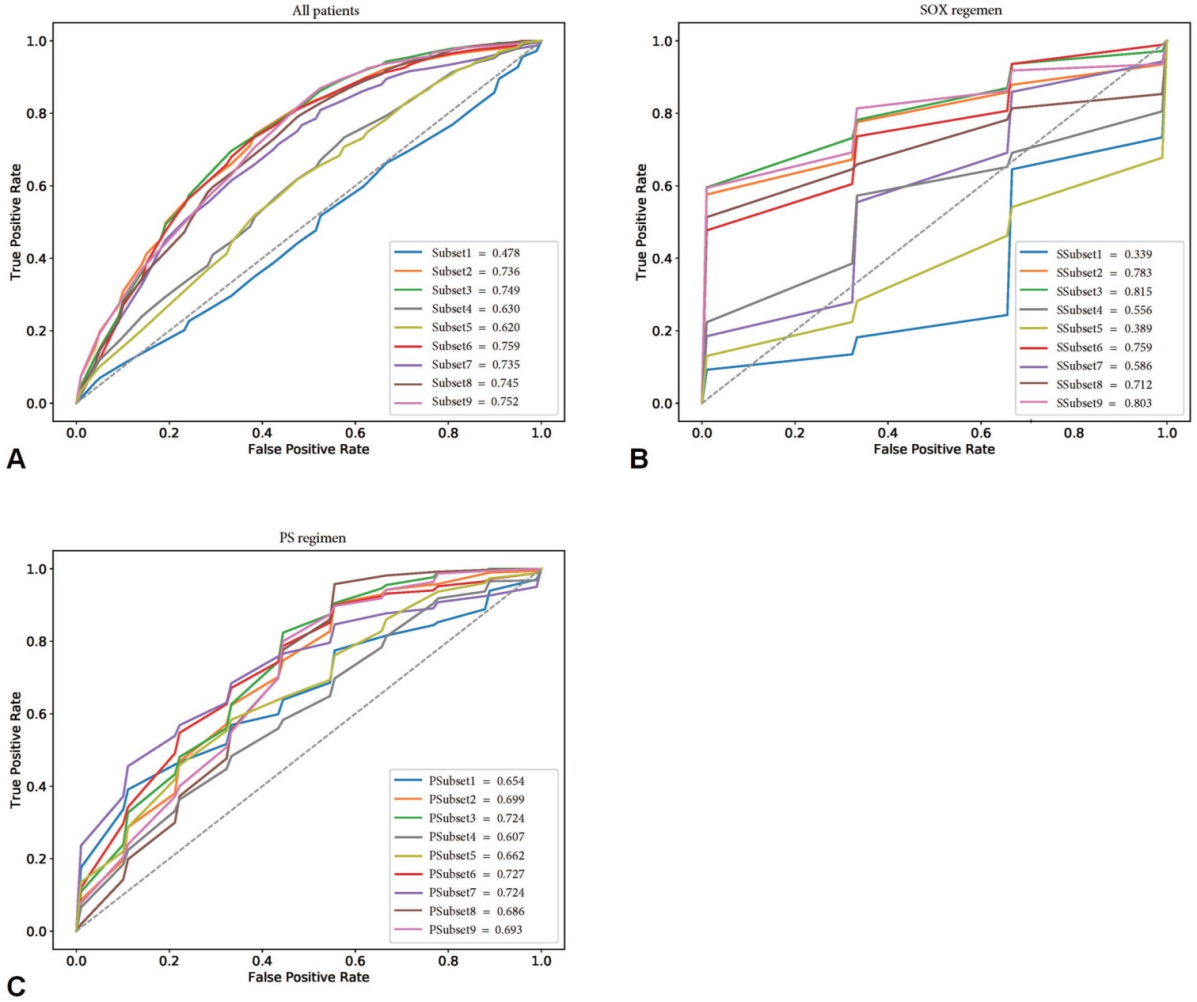
Receiver operating characteristic curves (ROCs) of nine feature subsets in the training cohorts. (A) showed the performance of nine feature subsets for all patients in the training cohort. (B) and (C) showed the performance of nine feature subsets for patients treated with SOX regimen and PS regimen in the training cohort, respectively.

This provided an interesting clue for further investigation of the featured characteristics of the corresponding specific chemotherapy regimen. Sensitivity analysis for the chemotherapeutic response showed that the SOX regimen performed the best in the training cohort, with the best subset being SSubset 9 (mean ACC and AUC of 80.0% and 0.803, respectively), which was consistent with the results for all patients. Interestingly, the optimal feature subset with the best performance for the PS regimen was PSubset 3. However, shape features seemed to be the most important, as PSubset 6 performed nearly the same as the former (mean ACC of 73.9% vs. 73.6% and mean AUC of 0.724 vs. 0.727). The results of the two regimens for all nine feature subsets are presented in Table S4, Table S5 and Figure 3.

### Performance of manual and semi-automatic segmentation in the testing cohort and the independent validation cohort

#### Effectiveness of the semi-automatic segmentation compared with manual segmentation in the testing cohort and the independent validation cohort

**Figure 4:**
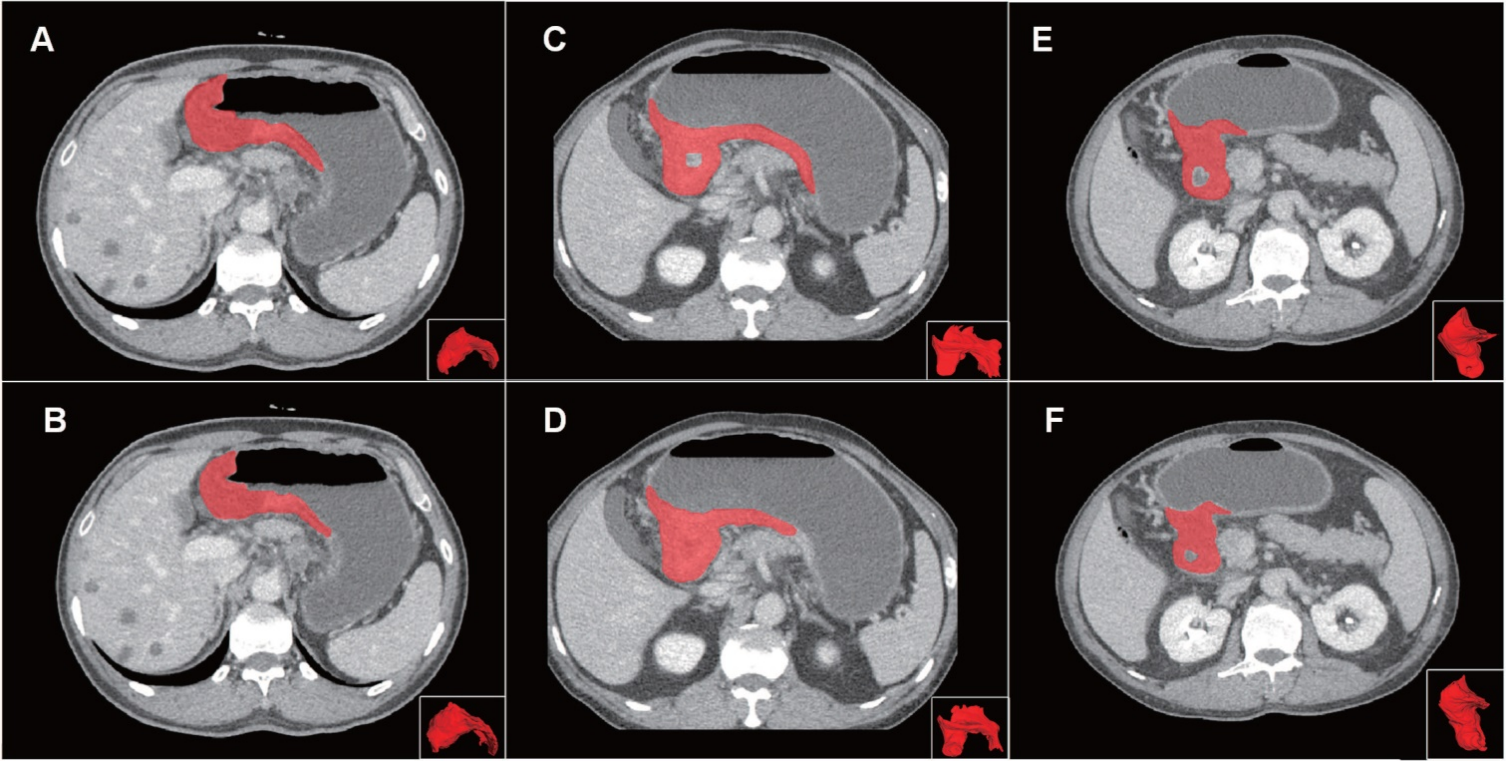
Examples of volumes of interest (VOIs) for comparison of manual and semi-automatic segmentation. Panel on the top (A, C, E) showed the reference of manual segmentation for three VOIs on axial images and 3D presentation. Panel on the below (B, D, F) showed the corresponding semi-automatic segmentation for each VOI. The VOIs from left to right severally presented good, moderate and poor Dice coefficients (0.857, 0.667 and 0.444) of the semi-automatic segmentation. Abbreviation: VOI: volume of interest.

**Figure 5:**
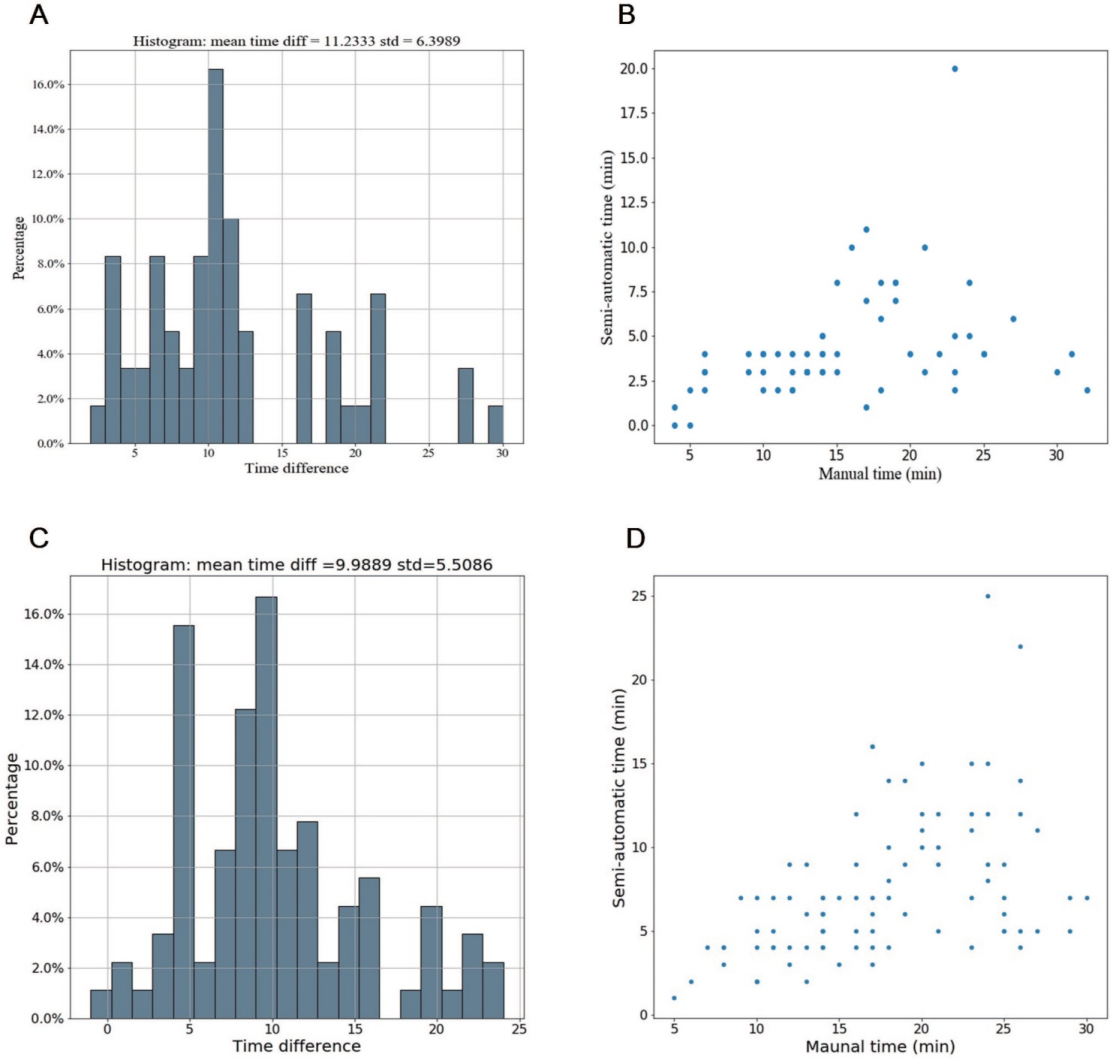
Time efficiency of the semi-automatic segmentation compared with manual segmentation in the testing cohort and the independent validation cohort. (A-B) Distribution of time efficiency for all VOIs of the semi-automatic segmentation compared with manual segmentation in the testing cohort presented in the histogram and scatter plot. The histogram showed the distribution of saving time. The scatter plot showed time difference of manual segmentation and semi-automatic segmentation for each VOI. (C-D) Distribution of time efficiency for all VOIs of the semi-automatic segmentation compared with manual segmentation in the indenpedent validation cohort presented in the histogram and scatter plot. Abbreviation: VOI: volume of interest.

Compared with manual segmentation, the semi-automatic segmentation was significantly more time-saving, with average saving-time of 11.2333 ± 6.3989 minutes and 9.9889 ±5.5086 minutes in the testing cohort and the independent validation cohort, respectively (both p < 0.05). The average Dice similarity coefficient for patients was 0.637 ± 0.213 and 0.618 ± 0.227 in the testing cohort and the independent validation cohort respectively, indicating a moderate performance of the semi-automatic segmentation (Figure 4 and Figure 5).

#### Predictive value of manual and semi-automatic segmentation methods for the chemotherapeutic response in the testing group

**Figure 6:**
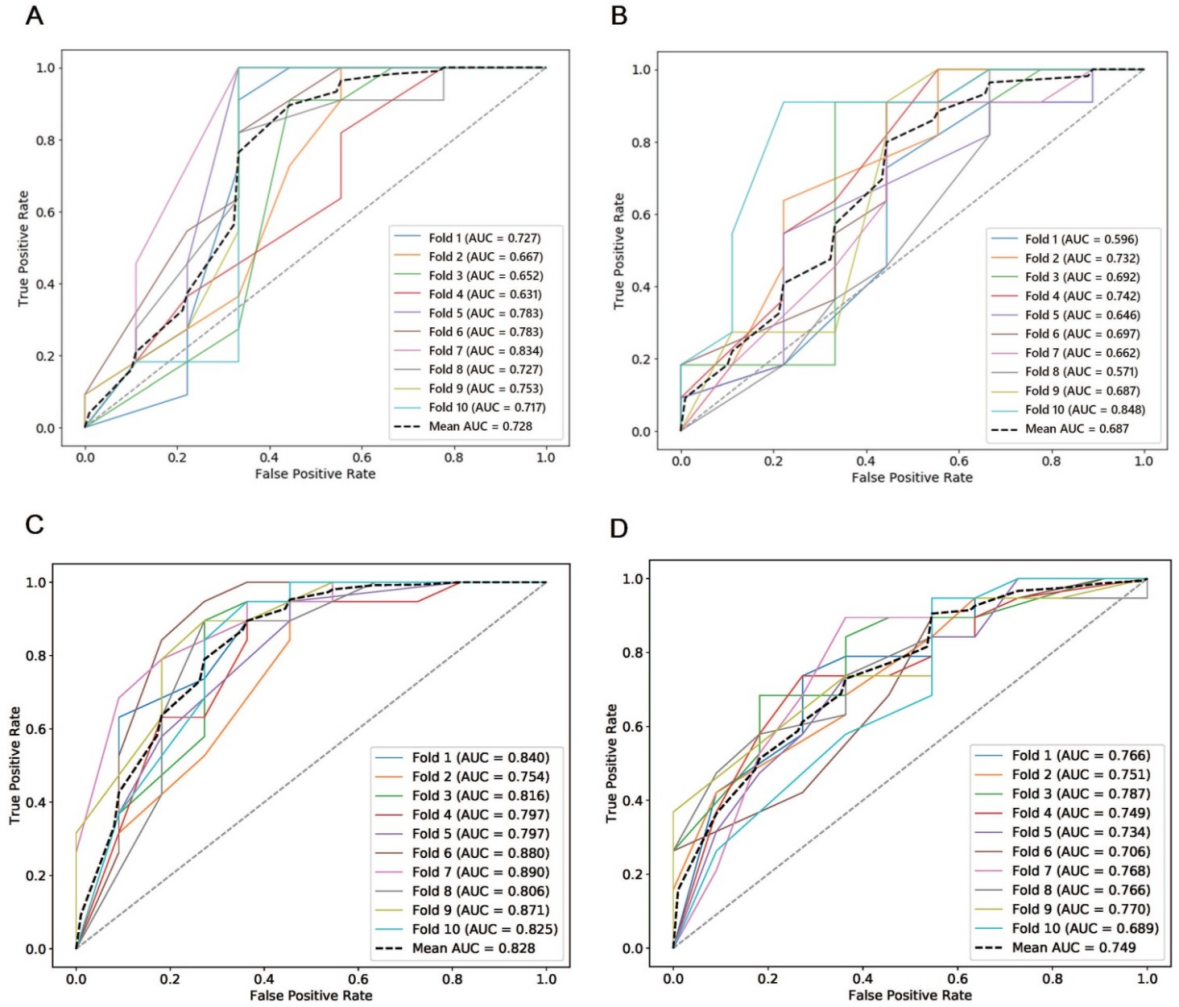
ROCs of semi-automatic segmentation and manual segmentation using 10-fold cross validation in the testing cohort and the independent validation cohort. (A) ROCs of semi-automatic segmentation using 10-fold cross validation in the testing cohort; (B) ROCs of manual segmentation using 10-fold cross validation in the testing cohort; (C) ROCs of semi-automatic segmentation using 10-fold cross validation in the independent validation cohort; (D) ROCs of manual segmentation using 10-fold cross validation in the independent validation cohort.

The combined total subset was considered the best feature subset and was used for predicting the chemotherapeutic response by both manual and semi-automatic segmentation in the testing cohort. The predictive value of the manual, combined model reached a mean AUC of 0.687 and 0.749 in the testing cohort and the independent validation cohort, respectively. The performance of the semi-automatic delta radiomics model was improved compared with manual segmentation, with a mean AUC of 0.728 and 0.828 in the testing cohort and the independent validation cohort, respectively. Detailed information is presented in Figure 6.

## Discussion

In this study, we developed and validated a DECT delta radiomics model to predict the treatment response at early stages of chemotherapy courses for far-advanced GC patients. Our delta radiomics model based on semi-automatic segmentation was not only more time-efficacy, but also performed better to predict the chemotherapeutic response for far-advanced GC patients than that of the manual segmentation. Proper intervention could be introduced to avoid unnecessary treatment or transfer to alternative therapeutic strategies using our delta radiomics model.

In 2011, delta radiomics was successfully used to predict the response of metastatic renal cell cancer to tyrosine kinase inhibitor treatment 30. Subsequently, delta radiomics showed predictive value for the chemotherapeutic response in colorectal liver metastases, rectal cancer and soft-tissue sarcoma 31-33. Our study is the first application of delta radiomics to predict the chemotherapeutic response of GC at succedent instead of current timepoints. Studies applying radiomics analysis to predict treatment response usually depend on baseline images, which may present with patient-specific differences and cannot reflect the dynamic changes in tumor response to treatment.

In contrast, delta radiomics could potentially minimize the baseline variance and reflect the dynamic changes of the tumor not only in terms of anatomy but also for intratumor heterogeneity. However, time intervals and maximum tumor length differences between different DECT scans could contribute to the variance in tumors among patients. Therefore, we independently analyzed time intervals and max tumor length to predict the chemotherapeutic response, which showed poor performance. When combining confounding factors and radiomics features for modeling, the combined model improved slightly compared with the model generated with radiomics features only. The results showed limited influence of time intervals and max tumor length for treatment response on follow-up. Notably, this is also the first delta radiomics model using dual-energy CT. Conventional CT, performed by single energy, is of limited value in differentiating tissues, irrespective of whether they have different elemental compositions. DECT, which simultaneously acquires dual-energy data, allows for distinguishing material-specific attenuation in composition and thus has the potential to provide more pathophysiological information 14. In a previous study, we found that total iodine uptake has predictive value for pathological regression in advanced GC after neoadjuvant chemotherapy 34. In this study, pathophysiological information, probably provided both by dual-energy CT and delta radiomics, contributes to the predictive value for the chemotherapeutic response.

Another important factor determining the difference in therapeutic response was the chemotherapeutic regimen. We performed a sensitivity analysis between two major chemotherapeutic regimens in the training cohort. Intriguingly, inconsistency was found for the two regimens. For the SOX regimen, the combined texture subset performed the best, while the shape subset performed best for the PS regimen. The underlying remedies for the SOX and PS regimens are oxaliplatin and paclitaxel, respectively. Oxaliplatin is a derivative of platinum, which is a type of cycle-independent drug that forms platinum-DNA adducts by covalently bonding with guanine and thus interferes with DNA replication, consequently resulting in the apoptosis of proliferating cells 35. The cumulative apoptosis of tumor cells may contribute to local necrosis inside tumors, and so it is reasonable that texture features reflect heterogeneity in this study. In contrast, paclitaxel is a well-known antitumor drug that targets tubulin. Paclitaxel protects microtubule polymers from disassembly by binding to beta-tubulin subunits, which are necessary for mitosis 36, 37. In this way, tumor cells are blocked from proliferation via defects in cell division. A deceased in the proliferation of tumor cells may account for changes in shape instead of heterogeneity.

Delineation of the entire tumor may allow the analysis of changes in the spatial variations of tumors more accurately than linear measurements 7, 38. However, the poor reproducibility and high labor costs from manual segmentation undoubtedly hinder the generalization of this method. In this study, we developed a semi-automatic segmentation method via a modified V-Net CNN deep learning algorithm, which yielded moderate reproducibility. Unlike its parent algorithm U-Net, another well-recognized CNN segmentation model, V-Net was designed specifically to delineate 3D regions 28. To our knowledge, no studies have delineated GC on CT images using deep learning. Fu et al 39 developed a CNN-based method to delineate abdominal organs for MRI-guided adaptive radiotherapy, and the Dice coefficient for the stomach in the study was 85.0 ± 3.75. Gibson and colleagues obtained a Dice coefficient of 0.90 for the stomach on abdominal CT with dense V-networks 40. A recent study used deep learning-based reiterative learning to perform a weakly supervised segmentation of GC applied to partially labelled pathological images, which yielded a union coefficient of 0.883 and a mean accuracy of 91.09% 20. In our study, the performance of our proposed semi-automatic method was moderate, with a mean Dice coefficient of 0.637 and 0.618 in the testing cohort and the independent validation cohort, respectively. For prudent reasons, we named our segmentation method semi-automatic instead of automatic, because more GC images on DECT are needed to improve the performance. Apart from the limited dataset, other possible reasons for the moderate Dice coefficient were the fickle appearance of GC tumors, especially after chemotherapy and in our experience, the relatively small lesions, which contributed to unsatisfactory recognition by our method. However, after manual calibration, our proposed semi-automatic segmentation was significantly less time-consuming compared with manual segmentation, saving more than 10 minutes for each VOI on average. Our semi-automatic segmentation is more effective than manual segmentation and has potential for clinical use in GC.

There are several limitations in our study. First, the sample size was small, both for the radiomics model and the deep learning-based semi-automatic segmentation. To minimize this deficiency, we adopted 10 rounds of 10-fold cross-validation, which was rigorous and not arbitrary to guarantee the reproducibility of our study. Furthermore, to train our V-net deep learning algorithm for volumetric segmentation, we augmented the original training dataset by rotating and warping the images. Second, this is a retrospective study from a single tertiary hospital, which may inevitably lead to selective bias for the patients. The results need to be validated by prospective and external cohorts. Third, due to performing the study during the early stage of the chemotherapy courses, there were no patients presenting with disease progression when evaluating the response, which may weaken the clinical use of our CT delta radiomics model for proper interventions. Last, although we performed a sensitivity analysis for two chemotherapy regimens that were most commonly used and the results suggested limited influence of the different drugs on the variance of treatment response, due to limited sample size, the performance of each chemotherapy regimen needs further elucidation.

## Conclusion

In conclusion, our proposed delta radiomics model serves as a promising method for determining biomarkers to predict the response during chemotherapy courses for far-advanced GC, providing potential and alternative interferences for further treatments. Moreover, the designed semi-automatic segmentation based on deep learning for tumor volume shows the potential for profound clinical use with increased reproducibility and decreased labor costs.

## Supplementary Material

Supplementary methods, figure and tables.

## Figures and Tables

**Table 1:** Clinical characteristics of the training and testing cohorts

	The training cohort			The testing cohort		
	Response group (n=21)	Non-response group (n=45)	*p*	Response group (n=9)	Non-response group (n=11)	*p*
Gender			0.045*			0.670
Male	9 (42.3%)	31 (68.9%)		6 (66.7%)	6 (54.5%)	
Female	12 (57.7%)	14 (31.1%)		3 (33.3%)	5 (45.5%)	
Age, y	57.19 ± 11.63	59.11 ± 12.72	0.560	55.44 ± 16.39	59.00 ± 7.38	0.526
Stage			0.216			1.000
Ⅲ	2 (9.5%)	10 (22.2%)		3 (33.3%)	4 (36.4%)	
Ⅳ	19 (90.5%)	35 (77.8%)		6 (66.7%)	7 (63.6%)	
Metastasis			0.454			1.000
Yes	3 (14.3%)	10 (22.2%)		3 (33.3%)	4 (36.4%)	
No	18 (85.7%)	35 (77.8%)		6 (66.7%)	7 (63.6%)	
Borrmann type			0.098			1.000
Type Ⅰ	1 (4.8%)	0 (0.0%)		0 (0.0%)	1 (9.1%)	
Type Ⅱ	2 (9.4%)	1 (2.2%)		0 (0.0%)	0 (0.0%)	
Type Ⅲ	17 (81.0%)	36 (80.0%)		9 (100%)	9 (81.8%)	
Type Ⅳ	1 (4.8%)	8 (17.8%)		0 (0.0%)	1 (9.1%)	
Tumor location			0.765			0.256
Upper	2 (9.5%)	6 (13.3%)		1 (11.1%)	1 (9.1%)	
Middle	2 (9.5%)	9 (20.0%)		1 (11.1%)	1 (9.1%)	
Lower	5 (23.8%)	9 (20.0%)		5 (55.6%)	2 (18.2%)	
Diffuse	12 (57.2%)	21 (46.7%)		2 (22.2%)	7 (63.6%)	

## Abbreviations

AUC: area under the curve
ACC: accuracy
CNN: convolutional neural network
CT: computed tomography
DECT: dual-energy computed tomography
GC: gastric cancer
GLCM: gray level co-occurrence matrix
GLDM: gray level dependence matrix
GLRLM: gray level run length matrix
GLSZM: gray level size zone matrix
MRI: magnetic resonance imaging
NGTDM: neighboring gray tone difference matrix
RECIST: response evaluation criteria in solid tumors
RF: random forest
ROC: receiver operating characteristic curve
ROI: region of interest
VOIs: volumes of interest (s)
