# Supplementary material for: Predicting Chemotherapeutic Response for Far-advanced Gastric Cancer by Radiomics with Deep Learning Semi-automatic Segmentation
Source: J Cancer. 2020 Oct 18;11(24):7224–36. doi: 10.7150/jca.46704 (PMC7646171; doi:10.7150/jca.46704)
Supplement: Supplementary file 1 — Supplementary methods, figure and tables. [file jcav11p7224s1.pdf]

Supplementary materials

Doc S1 Imaging protocol

All patients underwent dual-energy computed tomography by either of two scanners (Siemens SOMATOM Definition Flash or SOMATOM Force, Siemens Medical Solutions, Forchheim, Germany) after fasting overnight. Before the examination, all patients drank 1000-1500 ml of water and were injected with 20 mg of scopolamine. The tube voltages of the DECT scans were 100 kV and Sn140 kV, with reference tube currents of 230 mA and 178 mA, respectively. Real-time tube current modulation (CARE Dose 4D, Siemens Medical Solutions) was performed when scanning with a collimator of 32 × 0.6 mm and a pitch of 0.6. After a routine nonenhanced scan, three contrast-enhanced phases (arterial phase, portal venous phase and delayed phase) were performed following the intravenous administration of iodinated contrast material (Ultravist 370; Schering, Berlin, Germany) at 1.5 mL per kilogram at a rate of 3.5 mL/s using a pump injector. The arterial phase was determined by the time to peak enhancement of the celiac trunk, which covered the whole stomach. The portal venous phase and delayed phase then followed, with a delay time of 20 s after the arterial phase and 150 s after the administration of the contrast agents. All raw images were reconstructed by a D30f kernel and formed mixed 120 kV with a linear blending technique using a slice thickness of 1.5 mm. Then, all images were anonymously retrieved.

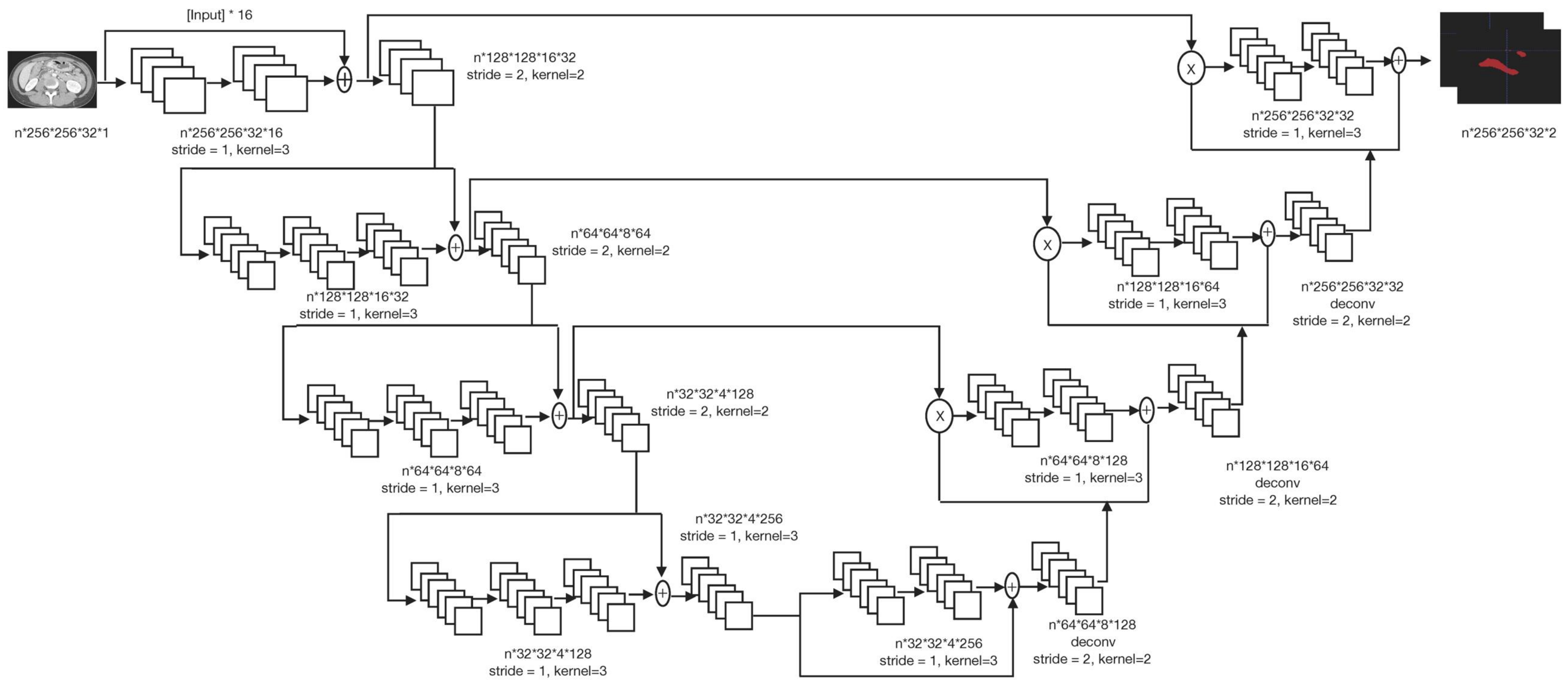

Figure S1 The structure of V-net for semi-automatic segmentation designed in this study

Table S1 Information for extracted radiomics features

Categories	Features	Definition
**First Order Features (**describe the distribution of voxel intensities within the image region defined by the mask through commonly used and basic metrics.)	**Energy**	Energy is a measure of the magnitude of voxel values in an image. A larger value implies a greater sum of the squares of these values.
	**Total Energy**	Total Energy is the value of Energy feature scaled by the volume of the voxel in cubic mm.
	**Entropy**	Entropy specifies the uncertainty/randomness in the image values. It measures the average amount of information required to encode the image values.
	**Minimum**	The minimum value of the voxel intensities
	**10th percentile**	The 10th percentile of the voxel intensities
	**90th percentile**	The 90th percentile of the voxel intensities
	**Maximum**	The maximum gray level intensity within the ROI.
	**Mean**	The average gray level intensity within the ROI.
	**Median**	The median gray level intensity within the ROI.
	**Interquartile Range**	The interquartile range of gray values in the ROI.
	**Range**	The range of gray values in the ROI.
	**Mean Absolute Deviation (MAD)**	Mean Absolute Deviation is the mean distance of all intensity values from the Mean Value of the image array.
	**Robust Mean Absolute Deviation (rMAD)**	Robust Mean Absolute Deviation is the mean distance of all intensity values from the Mean Value calculated on the subset of image array with gray levels in between, or equal to the 10th and 90th percentile.
	**Root Mean Squared (RMS)**	RMS is the square-root of the mean of all the squared intensity values. It is another measure of the magnitude of the image values. This feature is volume-confounded, a larger value of cc increases the effect of volume-confounding.
	**Skewness**	Skewness measures the asymmetry of the distribution of values about the Mean value. Depending on where the tail is elongated and the mass of the distribution is concentrated, this value can be positive

		or negative.
	**Kurtosis**	Kurtosis is a measure of the 'peak' of the distribution of values in the image ROI. A higher kurtosis implies that the mass of the distribution is concentrated towards the tail(s) rather than towards the mean. A lower kurtosis implies the reverse: that the mass of the distribution is concentrated towards a spike near the Mean value.
	**Variance**	Variance is the mean of the squared distances of each intensity value from the Mean value. This is a measure of the spread of the distribution about the mean.
	**Uniformity**	Uniformity is a measure of the sum of the squares of each intensity value. This is a measure of the homogeneity of the image array, where a greater uniformity implies a greater homogeneity or a smaller range of discrete intensity values.
**Shape Features (3D)** (In this group of features we included descriptors of the three-dimensional size and shape of the ROI. These features are independent from the gray level intensity distribution in the ROI and are therefore only calculated on the non-derived image and mask.)	**Mesh Volume**	The sign of the volume is determined by the sign of the normal, which must be consistently defined as either facing outward or inward of the ROI.
	**Voxel Volume**	This is a less precise approximation of the volume and is not used in subsequent features. This feature does not make use of the mesh and is not used in calculation of other shape features.
	**Surface Area**	The sum of all triangle areas in the mesh
	**Surface Area to Volume ratio**	Here, a lower value indicates a more compact (sphere-like) shape. This feature is not dimensionless, and is therefore (partly) dependent on the volume of the ROI.
	**Sphericity**	Sphericity is a measure of the roundness of the shape of the tumor region relative to a sphere. It is a dimensionless measure, independent of scale and orientation.
	**Maximum 3D diameter**	Maximum 3D diameter is defined as the largest pairwise Euclidean distance between tumor surface mesh vertices.
	**Maximum 2D diameter (Slice)**	Maximum 2D diameter (Slice) is defined as the largest pairwise Euclidean distance between tumor surface mesh vertices in the row-column (generally the axial) plane.

	**Maximum 2D diameter (Column)**	Maximum 2D diameter (Column) is defined as the largest pairwise Euclidean distance between tumor surface mesh vertices in the row-slice (usually the coronal) plane.
	**Maximum 2D diameter (Row)**	Maximum 2D diameter (Row) is defined as the largest pairwise Euclidean distance between tumor surface mesh vertices in the column-slice (usually the sagittal) plane.
	**Major Axis Length**	This feature yields the largest axis length of the ROI-enclosing ellipsoid and is calculated using the largest principal component λ major.
	**Minor Axis Length**	This feature yields the second-largest axis length of the ROI-enclosing ellipsoid and is calculated using the largest principal component λ minor.
	**Least Axis Length**	This feature yields the smallest axis length of the ROI-enclosing ellipsoid and is calculated using the largest principal component λ least. In case of a 2D segmentation, this value will be 0.
	**Elongation**	Elongation shows the relationship between the two largest principal components in the ROI shape. For computational reasons, this feature is defined as the inverse of true elongation.
	**Flatness**	Flatness shows the relationship between the largest and smallest principal components in the ROI shape. For computational reasons, this feature is defined as the inverse of true flatness.
**Gray Level Co-occurrence Matrix (GLCM) Features** (A Gray Level Co-occurrence Matrix (GLCM) describes the second-order joint probability function of an image region constrained by the mask)	**Autocorrelation**	Autocorrelation is a measure of the magnitude of the fineness and coarseness of texture.
	**Joint Average**	Returns the mean gray level intensity of the ii distribution.
	**Cluster Prominence**	Cluster Prominence is a measure of the skewness and asymmetry of the GLCM. A higher value implies more asymmetry about the mean while a lower value indicates a peak near the mean value and less variation about the mean.
	**Cluster Shade**	Cluster Shade is a measure of the skewness and uniformity of the GLCM. A higher cluster shade implies greater asymmetry about the mean.

	**Cluster Tendency**	Cluster Tendency is a measure of groupings of voxels with similar gray-level values.
	**Contrast**	Contrast is a measure of the local intensity variation, favoring values away from the diagonal. A larger value correlates with a greater disparity in intensity values among neighboring voxels.
	**Correlation**	Correlation is a value between 0 (uncorrelated) and 1 (perfectly correlated) showing the linear dependency of gray level values to their respective voxels in the GLCM.
	**Difference Average**	Difference Average measures the relationship between occurrences of pairs with similar intensity values and occurrences of pairs with differing intensity values.
	**Difference Entropy**	Difference Entropy is a measure of the randomness/variability in neighborhood intensity value differences.
	**Difference Variance**	Difference Variance is a measure of heterogeneity that places higher weights on differing intensity level pairs that deviate more from the mean.
	**Joint Energy**	Energy is a measure of homogeneous patterns in the image. A greater Energy implies that there are more instances of intensity value pairs in the image that neighbor each other at higher frequencies.
	**Joint Entropy**	Joint entropy is a measure of the randomness/variability in neighborhood intensity values.
	**Informational Measure of Correlation (IMC) 1**	IMC1 assesses the correlation between the probability distributions of ii and jj (quantifying the complexity of the texture), using mutual information.
	**Informational Measure of Correlation (IMC) 2**	IMC2 also assesses the correlation between the probability distributions of ii and jj (quantifying the complexity of the texture).
	**Inverse Difference Moment (IDM)**	IDM (a.k.a Homogeneity 2) is a measure of the local homogeneity of an image. IDM weights are the inverse of the Contrast weights (decreasing exponentially from the diagonal in the GLCM).
	**Maximal Correlation Coefficient**	The Maximal Correlation Coefficient is a measure of complexity of the texture and $0 \leq MCC \leq 1$.

	**(MCC)**	
	**Inverse Difference Moment Normalized (IDMN)**	IDMN (inverse difference moment normalized) is a measure of the local homogeneity of an image. IDMN weights are the inverse of the Contrast weights (decreasing exponentially from the diagonal in the GLCM).
	**Inverse Difference (ID)**	ID (a.k.a. Homogeneity 1) is another measure of the local homogeneity of an image. With more uniform gray levels, the denominator will remain low, resulting in a higher overall value.
	**Inverse Difference Normalized (IDN)**	IDN (inverse difference normalized) is another measure of the local homogeneity of an image. Unlike Homogeneity1, IDN normalizes the difference between the neighboring intensity values by dividing over the total number of discrete intensity values.
	**Inverse Variance**	The inverse variance is another measure of the local homogeneity of an image.
	**Maximum Probability**	Maximum Probability is occurrences of the most predominant pair of neighboring intensity values.
	**Sum Average**	Sum Average measures the relationship between occurrences of pairs with lower intensity values and occurrences of pairs with higher intensity values.
	**Sum Entropy**	Sum Entropy is a sum of neighborhood intensity value differences.
	**Sum of Squares**	Sum of Squares or Variance is a measure in the distribution of neighboring intensity level pairs about the mean intensity level in the GLCM.
**Gray Level Size Zone Matrix (GLSZM) Features** (A Gray Level Size Zone (GLSZM) quantifies gray level zones in an image. A gray level zone is defined as the	**Small Area Emphasis (SAE)**	SAE is a measure of the distribution of small size zones, with a greater value indicative of more smaller size zones and more fine textures.
	**Large Area Emphasis (LAE)**	LAE is a measure of the distribution of large area size zones, with a greater value indicative of more larger size zones and more coarse textures.
	**Gray Level Non-Uniformity (GLN)**	GLN measures the variability of gray-level intensity values in the image, with a lower value indicating more homogeneity in intensity values.

number of connected voxels that share the same gray level intensity.)	**Gray Level Non-Uniformity Normalized (GLNN)**	GLNN measures the variability of gray-level intensity values in the image, with a lower value indicating a greater similarity in intensity values. This is the normalized version of the GLN formula.
	**Size-Zone Non-Uniformity (SZN)**	SZN measures the variability of size zone volumes in the image, with a lower value indicating more homogeneity in size zone volumes.
	**Size-Zone Non-Uniformity Normalized (SZNN)**	SZNN measures the variability of size zone volumes throughout the image, with a lower value indicating more homogeneity among zone size volumes in the image. This is the normalized version of the SZN formula.
	**Zone Percentage (ZP)**	ZP measures the coarseness of the texture by taking the ratio of number of zones and number of voxels in the ROI.
	**Gray Level Variance (GLV)**	GLV measures the variance in gray level intensities for the zones.
	**Zone Variance (ZV)**	ZV measures the variance in zone size volumes for the zones.
	**Zone Entropy (ZE)**	ZE measures the uncertainty/randomness in the distribution of zone sizes and gray levels. A higher value indicates more heterogeneity in the texture patterns.
	**Low Gray Level Zone Emphasis (LGLZE)**	LGLZE measures the distribution of lower gray-level size zones, with a higher value indicating a greater proportion of lower gray-level values and size zones in the image.
	**High Gray Level Zone Emphasis (HGLZE)**	HGLZE measures the distribution of the higher gray-level values, with a higher value indicating a greater proportion of higher gray-level values and size zones in the image.
	**Small Area Low Gray Level Emphasis (SALGLE)**	SALGLE measures the proportion in the image of the joint distribution of smaller size zones with lower gray-level values.
	**Small Area High Gray Level Emphasis (SAHGLE)**	SAHGLE measures the proportion in the image of the joint distribution of smaller size zones with higher gray-level values.
	**Large Area Low Gray Level Emphasis (LALGLE)**	LALGLE measures the proportion in the image of the joint distribution of larger size zones with lower gray-level values.

	**Large Area High Gray Level Emphasis (LAHGLE)**	LAHGLE measures the proportion in the image of the joint distribution of larger size zones with higher gray-level values.
**Gray Level Run Length Matrix (GLRLM) Features** (A Gray Level Run Length Matrix (GLRLM) quantifies gray level runs, which are defined as the length in number of pixels, of consecutive pixels that have the same gray level value.)	**Short Run Emphasis (SRE)**	SRE is a measure of the distribution of short run lengths, with a greater value indicative of shorter run lengths and more fine textural textures.
	**Long Run Emphasis (LRE)**	LRE is a measure of the distribution of long run lengths, with a greater value indicative of longer run lengths and more coarse structural textures.
	**Gray Level Non-Uniformity (GLN)**	GLN measures the similarity of gray-level intensity values in the image, where a lower GLN value correlates with a greater similarity in intensity values.
	**Gray Level Non-Uniformity Normalized (GLNN)**	GLNN measures the similarity of gray-level intensity values in the image, where a lower GLNN value correlates with a greater similarity in intensity values. This is the normalized version of the GLN formula.
	**Run Length Non-Uniformity (RLN)**	RLN measures the similarity of run lengths throughout the image, with a lower value indicating more homogeneity among run lengths in the image.
	**Run Length Non-Uniformity Normalized (RLNN)**	RLNN measures the similarity of run lengths throughout the image, with a lower value indicating more homogeneity among run lengths in the image. This is the normalized version of the RLN formula.
	**Run Percentage (RP)**	RP measures the coarseness of the texture by taking the ratio of number of runs and number of voxels in the ROI.
	**Gray Level Variance (GLV)**	GLV measures the variance in gray level intensity for the runs.
	**Run Variance (RV)**	RV is a measure of the variance in runs for the run lengths.
	**Run Entropy (RE)**	RE measures the uncertainty/randomness in the distribution of run lengths and gray levels. A higher value indicates more heterogeneity in the texture patterns.
	**Low Gray Level Run Emphasis (LGLRE)**	LGLRE measures the distribution of low gray-level values, with a higher value indicating a greater concentration of low gray-level values in the image.

	**High Gray Level Run Emphasis (HGLRE)**	HGLRE measures the distribution of the higher gray-level values, with a higher value indicating a greater concentration of high gray-level values in the image.
	**Short Run Low Gray Level Emphasis (SRLGLE)**	SRLGLE measures the joint distribution of shorter run lengths with lower gray-level values.
	**Short Run High Gray Level Emphasis (SRHGLE)**	SRHGLE measures the joint distribution of shorter run lengths with higher gray-level values.
	**Long Run Low Gray Level Emphasis (LRLGLE)**	LRLGLRE measures the joint distribution of long run lengths with lower gray-level values.
	**Long Run High Gray Level Emphasis (LRHGLE)**	LRHGLRE measures the joint distribution of long run lengths with higher gray-level values.
**Neighboring Gray Tone Difference Matrix (NGTDM) Features** (A Neighboring Gray Tone Difference Matrix quantifies the difference between a gray value and the average gray value of its neighbors within distance)	**Coarseness**	Coarseness is a measure of average difference between the center voxel and its neighborhood and is an indication of the spatial rate of change. A higher value indicates a lower spatial change rate and a locally more uniform texture.
	**Contrast**	Contrast is a measure of the spatial intensity change, but is also dependent on the overall gray level dynamic range. Contrast is high when both the dynamic range and the spatial change rate are high, i.e. an image with a large range of gray levels, with large changes between voxels and their neighborhood.
	**Busyness**	A measure of the change from a pixel to its neighbor. A high value for busyness indicates a ‘busy’ image, with rapid changes of intensity between pixels and its neighborhood.
	**Complexity**	An image is considered complex when there are many primitive components in the image, i.e. the image is non-uniform and there are many rapid changes in gray level intensity.
	**Strength**	Strength is a measure of the primitives in an image. Its value is high when the primitives are easily defined and visible, i.e. an image with slow change in intensity but more large coarse differences in gray level intensities.

**Gray Level Dependence Matrix (GLDM) Features** (A Gray Level Dependence Matrix (GLDM) quantifies gray level dependencies in an image.)	**Small Dependence Emphasis (SDE)**	A measure of the distribution of small dependencies, with a greater value indicative of smaller dependence and less homogeneous textures.
	**Large Dependence Emphasis (LDE)**	A measure of the distribution of large dependencies, with a greater value indicative of larger dependence and more homogeneous textures.
	**Gray Level Non-Uniformity (GLN)**	Measures the similarity of gray-level intensity values in the image, where a lower GLN value correlates with a greater similarity in intensity values.
	**Dependence Non-Uniformity (DN)**	Measures the similarity of dependence throughout the image, with a lower value indicating more homogeneity among dependencies in the image.
	**Dependence Non-Uniformity Normalized (DNN)**	Measures the similarity of dependence throughout the image, with a lower value indicating more homogeneity among dependencies in the image. This is the normalized version of the DLN formula.
	**Gray Level Variance (GLV)**	Measures the variance in grey level in the image.
	**Dependence Variance (DV)**	Measures the variance in dependence size in the image.
	**Dependence Entropy (DE)**	
	**Low Gray Level Emphasis (LGLE)**	Measures the distribution of low gray-level values, with a higher value indicating a greater concentration of low gray-level values in the image.
	**High Gray Level Emphasis (HGLE)**	Measures the distribution of the higher gray-level values, with a higher value indicating a greater concentration of high gray-level values in the image.
	**Small Dependence Low Gray Level Emphasis (SDLGLE)**	Measures the joint distribution of small dependence with lower gray-level values.
	**Small Dependence High Gray Level Emphasis (SDHGLE)**	Measures the joint distribution of small dependence with higher gray-level values.
	**Large Dependence Low Gray Level Emphasis**	Measures the joint distribution of large dependence with lower gray-level values.

	**(LDLGLE)**	
	**Large Dependence High Gray Level Emphasis (LDHGLE)**	Measures the joint distribution of large dependence with higher gray-level values.

Table S2 Names of feature subsets for patients in the training cohort

	All patients	SOX regimen	PS regimen
Four confounding factors	Subset 1	SSubset 1	PSubset 1
All delta radiomics features	Subset 2	SSubset 2	PSubset 2
Combination of confounding factors and delta radiomics features	Subset 3	SSubset 3	PSubset 3
First-order features	Subset 4	SSubset 4	PSubset 4
Combination of confounding factors and first-order features	Subset 5	SSubset 5	PSubset 5
Shape features	Subset 6	SSubset 6	PSubset 6
Combination of confounding factors and shape features	Subset 7	SSubset 7	PSubset 7
Texture features	Subset 8	SSubset 8	PSubset 8
Combination of confounding factors and texture features	Subset 9	SSubset 9	PSubset 9

Table S3 Performance of nine feature subsets for all patients in the training cohort

Feature subset	Metrics	1	2	3	4	5	6	7	8	9	10	mean	std
Subset 1	ACC (%)	48.8	63.8	52.1	53.3	65.7	62.1	56.4	56.0	58.6	57.1	57.4	5.1
	AUC	0.408	0.661	0.463	0.468	0.447	0.370	0.522	0.455	0.437	0.550	0.478	0.078
Subset 2	ACC (%)	67.9	69.3	72.9	71.0	78.3	65.0	74.3	73.3	73.6	75.5	72.1	3.7
	AUC	0.662	0.767	0.729	0.703	0.791	0.698	0.789	0.756	0.731	0.730	0.736	0.039
Subset 3	ACC (%)	71.9	73.8	71.0	78.3	71.0	71.2	70.2	72.9	74.0	75.2	**73.0**	2.4
	AUC	0.708	0.783	0.719	0.839	0.773	0.730	0.760	0.747	0.682	0.745	**0.749**	0.042
Subset 4	ACC (%)	76.0	71.9	59.0	61.0	66.9	66.7	68.3	68.3	61.7	62.1	66.2	5.0
	AUC	0.749	0.692	0.647	0.544	0.577	0.680	0.617	0.543	0.652	0.598	0.630	0.063
Subset 5	ACC (%)	61.9	68.3	66.2	64.3	65.0	73.1	63.6	56.7	66.7	61.7	64.8	4.1
	AUC	0.648	0.663	0.668	0.528	0.683	0.657	0.586	0.505	0.645	0.621	0.620	0.058
Subset 6	ACC (%)	67.4	74.0	68.3	66.9	72.6	71.7	74.8	72.4	66.4	71.4	70.6	2.9
	AUC	0.760	0.763	0.787	0.808	0.760	0.766	0.749	0.715	0.767	0.710	0.759	0.028
Subset 7	ACC (%)	75.0	72.9	71.2	64.8	64.5	60.7	71.2	71.4	63.6	69.5	68.5	4.5
	AUC	0.724	0.753	0.764	0.762	0.633	0.772	0.740	0.717	0.665	0.816	0.735	0.051
Subset 8	ACC (%)	77.1	77.1	73.8	68.8	77.4	68.3	73.6	72.1	68.6	69.5	72.6	3.5
	AUC	0.824	0.828	0.805	0.675	0.865	0.699	0.720	0.677	0.675	0.678	0.745	0.073
Subset 9	ACC (%)	75.7	71.4	76.0	72.6	73.3	71.7	76.0	75.7	70.0	75.5	**73.8**	2.1
	AUC	0.798	0.786	0.818	0.768	0.748	0.666	0.736	0.698	0.776	0.725	**0.752**	0.044

Abbreviations: accuracy, ACC; area under the curve, AUC.

Table S4 Performance of nine feature subsets for patients with SOX regimen in the training cohort

Feature subset	Metrics	1	2	3	4	5	6	7	8	9	10	mean	std
SSubset 1	ACC (%)	42.9	50.0	42.9	57.1	71.4	71.4	50.0	57.1	78.6	64.3	58.6	11.9
	AUC	0.470	0.379	0.242	0.303	0.409	0.318	0.212	0.273	0.318	0.470	0.339	0.085
SSubset 2	ACC (%)	71.4	64.3	71.4	78.6	85.7	57.1	71.4	78.6	78.6	78.6	73.6	7.9
	AUC	0.788	0.773	0.803	0.697	0.864	0.697	0.788	0.833	0.788	0.803	0.783	0.050
SSubset 3	ACC (%)	78.6	78.6	85.7	71.4	78.6	64.3	64.3	78.6	78.6	78.6	75.0	6.6
	AUC	0.818	0.848	0.970	0.773	0.864	0.712	0.576	0.879	0.818	0.848	0.815	0.105
SSubset 4	ACC (%)	78.6	64.3	57.1	64.3	64.3	71.4	78.6	71.4	64.3	71.4	68.6	6.6
	AUC	0.621	0.742	0.606	0.470	0.364	0.439	0.712	0.621	0.470	0.515	0.556	0.117
SSubset 5	ACC (%)	78.6	71.4	78.6	57.1	64.3	57.1	57.1	42.9	64.3	57.1	62.9	10.5
	AUC	0.409	0.470	0.409	0.182	0.379	0.167	0.515	0.379	0.394	0.576	0.389	0.123
SSubset 6	ACC (%)	64.3	78.6	64.3	57.1	71.4	71.4	92.9	78.6	71.4	64.3	71.4	9.6
	AUC	0.712	0.833	0.652	0.697	0.742	0.742	0.939	0.667	0.848	0.758	0.759	0.085
SSubset 7	ACC (%)	64.3	57.1	71.4	57.1	57.1	64.3	71.4	71.4	50.0	50.0	61.4	8.0
	AUC	0.561	0.682	0.727	0.394	0.591	0.545	0.606	0.606	0.470	0.682	0.586	0.096
SSubset 8	ACC (%)	57.1	85.7	64.3	78.6	78.6	64.3	71.4	71.4	50.0	71.4	69.3	10.1
	AUC	0.576	0.939	0.727	0.788	0.803	0.682	0.652	0.758	0.500	0.697	0.712	0.117
SSubset 9	ACC (%)	85.7	71.4	71.4	78.6	85.7	71.4	92.9	78.6	85.7	78.6	**80.0**	**7.0**
	AUC	0.758	0.864	0.712	0.667	0.848	0.742	0.848	0.848	0.879	0.864	**0.803**	**0.072**

Abbreviations: accuracy, ACC; area under the curve, AUC.

Table S5 Performance of nine feature subsets for patients with PS regimen in the training cohort

Feature subset	Metrics	1	2	3	4	5	6	7	8	9	10	mean	std
PSubset 1	ACC (%)	57.1	75.0	67.9	60.7	60.7	64.3	64.3	71.4	67.9	67.9	65.7	5.1
	AUC	0.532	0.801	0.608	0.594	0.588	0.418	0.804	0.746	0.646	0.801	0.654	0.125
PSubset 2	ACC (%)	71.4	64.3	71.4	71.4	78.6	71.4	71.4	67.9	71.4	78.6	71.8	4.2
	AUC	0.740	0.649	0.637	0.751	0.751	0.687	0.719	0.658	0.667	0.734	0.699	0.042
PSubset 3	ACC (%)	71.4	82.1	67.9	82.1	64.3	67.9	71.4	71.4	82.1	78.6	73.9	6.4
	AUC	0.760	0.711	0.614	0.904	0.737	0.678	0.711	0.699	0.708	0.716	0.724	0.070
PSubset 4	ACC (%)	71.4	71.4	57.1	64.3	64.3	60.7	64.3	60.7	64.3	64.3	64.3	4.2
	AUC	0.588	0.643	0.614	0.526	0.544	0.667	0.696	0.538	0.673	0.585	0.607	0.058
PSubset 5	ACC (%)	71.4	64.3	67.9	67.9	67.9	85.7	71.4	60.7	71.4	64.3	69.3	6.4
	AUC	0.702	0.652	0.728	0.611	0.687	0.769	0.699	0.523	0.728	0.523	0.662	0.081
PSubset 6	ACC (%)	82.1	78.6	71.4	67.9	78.6	75.0	75.0	71.4	64.3	71.4	73.6	5.1
	AUC	0.863	0.687	0.754	0.757	0.778	0.681	0.626	0.713	0.696	0.716	0.727	0.062
PSubset 7	ACC (%)	67.9	75.0	71.4	71.4	67.9	57.1	71.4	64.3	67.9	75.0	68.9	5.1
	AUC	0.719	0.640	0.746	0.705	0.693	0.658	0.713	0.699	0.813	0.857	0.724	0.063
PSubset 8	ACC (%)	85.7	75.0	78.6	67.9	82.1	75.0	75.0	75.0	71.4	75.0	76.1	4.8
	AUC	0.687	0.740	0.757	0.649	0.798	0.699	0.637	0.623	0.658	0.608	0.686	0.059
PSubset 9	ACC (%)	78.6	64.3	75.0	71.4	78.6	78.6	78.6	71.4	64.3	75.0	73.6	5.4
	AUC	0.775	0.640	0.675	0.687	0.751	0.772	0.687	0.664	0.617	0.661	0.693	0.052

Abbreviations: accuracy, ACC; area under the curve, AUC.
